# Integrating biological data – the Distributed Annotation System

**DOI:** 10.1186/1471-2105-9-S8-S3

**Published:** 2008-07-22

**Authors:** Andrew M Jenkinson, Mario Albrecht, Ewan Birney, Hagen Blankenburg, Thomas Down, Robert D Finn, Henning Hermjakob, Tim JP Hubbard, Rafael C Jimenez, Philip Jones, Andreas Kähäri, Eugene Kulesha, José R Macías, Gabrielle A Reeves, Andreas Prlić

**Affiliations:** 1 European Bioinformatics Institute, Hinxton, Cambridge, CB10 1SD, UK; 2 Wellcome Trust Sanger Institute, Hinxton, Cambridge, CB10 1SA, UK; 3 Max Planck Institute for Informatics, Saarbrücken, 66123, Germany; 4 National Centre for Biotechnology, Madrid, 28049, Spain; 5 National Bioinformatics Network, Cape Town, 7405, South Africa; 6 Wellcome Trust/Cancer Research UK Gurdon Institute, University of Cambridge, Cambridge, CB2 1QN, UK

## Abstract

**Background:**

The Distributed Annotation System (DAS) is a widely adopted protocol for dynamically integrating a wide range of biological data from geographically diverse sources. DAS continues to expand its applicability and evolve in response to new challenges facing integrative bioinformatics.

**Results:**

Here we describe the various infrastructure components of DAS and present a new extended version of the DAS specification. Version 1.53E incorporates several recent developments, including its extension to serve new data types and an ontology for protein features.

**Conclusion:**

Our extensions to the DAS protocol have facilitated the integration of new data types, and our improvements to the existing DAS infrastructure have addressed recent challenges. The steadily increasing numbers of available data sources demonstrates further adoption of the DAS protocol.

## Background

The abundance of data in the post-genomics era is a major boon for life science researchers. However, data from disparate sources arguably have the most value when considered in context with each other. For example, manually curated experimental evidence may be more reliable than computational predictions, but the latter may offer greater coverage. Whilst drawing conclusions based on the results of multiple experiments is by no means a new concept in biology, *omics *data and *in silico *analyses make traditional *ad hoc *methods of publishing and sharing data impractical. With the trend for data expansion set to continue and the highly collaborative approaches of major projects such as ENCODE [1], integration is likely to become an increasingly important focus of bioinformatics.

Efforts to integrate data sources may be broadly categorised by their motivation:

1. aggregating and presenting data in an accessible format

2. computational analysis of combined data sets

3. federation of disparate resources

Each of these goals, although not necessarily mutually exclusive, has its own requirements. For example, whilst user interfaces must be responsive and accessible, computational analysis requires robust semantics.

### DAS overview

The Distributed Annotation System (DAS) [2] was originally conceived as a mechanism to aggregate and display genome sequence annotations such as transcript predictions. It is built upon the principle that data should remain spread across multiple sites, rather than aggregated into centralised databases. Thus data providers retain control over data access, releases can be more dynamic and changes to file formats or database structures are transparent. DAS has a "dumb server, clever client" architecture, which holds a number of advantages. For example, the minimal resources and time required of data providers to expose their data means more sources can be integrated and more readily. Conversely, one of the main reasons for this ease of implementation is a lack of enforced semantics, which limits applications primarily to visual display. In addition, DAS has been lacking a central registry of available data sources.

DAS was developed by WormBase [3] for sharing genome annotations, and was adopted by the Ensembl project [4] to facilitate the display of such distributed data in its genome browser. The applicability of DAS was extended to protein sequence and structure data by the efforts of the eFamily project to integrate five of the major protein databases [5,6]. It was subsequently adopted by the BioSapiens Network of Excellence as the mechanism of sharing proteomics data among member institutions [7,8], and also by the ENCODE project to dynamically share the latest data between collaborators. Many other individual projects across the world also expose their data and/or operate integration services via DAS.

As a standard for the sharing of biological information, the DAS protocol defines how data should be represented and communicated. It takes the form of a web service based upon the open standards of Hyper-Text Transfer Protocol (HTTP) for data transmission and Extensible Markup Language (XML) for data format. A DAS server may host a number of sources, each differing in the services it provides and the type of underlying data it is based on.

### Coordinate systems

DAS may be used to annotate different types of data. In order to distinguish these, *coordinate systems *describe the various reference data types DAS supports. Each coordinate system may be thought of as a model that bioinformaticians commonly use to denote biological entities and locations of features within them. A coordinate system has four parts:

1. The **category **or type of annotatable entity. For example a chromosome, gene, protein sequence or protein structure.

2. The **authority **or project responsible for defining the coordinate system. For example NCBI, UniProt or Ensembl.

3. The **version**, used where entities themselves are not versioned (as in genomic assemblies).

4. The **species**, for coordinate systems containing only entities from a single organism.

Though coordinate systems are normally used to describe the location of a feature within a reference entity (for example residue 26 of UniProt sequence P15056), some annotations are not always associated with a sequence location but rather the entity itself (for example database cross-references). Such features are commonly called *non-positional features *and are used most when annotating genes, which themselves are often thought of as abstract entities. The difference between annotating an entity versus a region of an entity's sequence is conceptual and requires no special implementation for a data source, but does have implications for a client's display.

### DAS commands

A DAS source may offer one or more different services to clients, determined by the *commands *it implements. A DAS command is a request issued by a client for a certain class of data, such as a sequence or annotations of a sequence. The server responds with an XML document representing the requested data. DAS defines a model for constructing the query (a specific URL format), a model for representing the data (an XML document type) and its means of transport (HTTP). Each command has similar but distinct query and data models. Version 1.53 of the DAS specification [9] has five main commands:

1. **entry points **– fetches a list of entities a source can annotate

2. **sequence **– fetches the sequence of a segment of DNA, protein *et cetera*

3. **features **– the most commonly implemented command; fetches annotations located within a segment

4. **types **– fetches a list of the types of feature a source or segment has

5. **stylesheet **– fetches instructions for displaying features

DAS sources that offer sequences are often referred to as *reference sources *because they provide the reference entry points for other commands on the same or different servers. Sources implementing the features command are by contrast referred to as *annotation sources *because they provide annotations based on a reference sequence. This distinction is largely historical since some DAS sources are conceptually both reference and annotation sources, and DAS has since expanded to cover non-sequence data.

The DAS specification has also been extended with several other commands, such as those offering 3D structures and alignments. These are discussed in the Results section.

### DAS registry

The steady growth in both the number and diversity of publicly available DAS sources necessitated the development of a method for the discovery of DAS services. Previously reported is the implementation of such a mechanism in the form of the DAS Registry [6,10]. This service allows data providers to publish their DAS sources, allowing their automatic discovery by compatible clients. This discovery feature has been incorporated into most client implementations and libraries. The registry also performs service validation on registered sources to check that they are both functioning and conforming to the DAS specification. The number of registered sources has steadily increased since the DAS registry was created, to date totalling 383.

## Results

In recent years the DAS protocol has been expanded beyond the core specification to cater for the data integration needs of additional areas of biological research. However these extensions have yet to be incorporated into the specification itself, the latest version of which is 1.53. Instead, collectively they form an extended version of the DAS protocol, version *1.53E*. This protocol, documented at , comprises five additional commands, an ontology for protein features, a server-side data preparation option (binning) and additional options for stylesheets. The extensions it offers are all optional for both servers and clients.

### New commands

The DAS 1.53E specification defines five new commands.

#### Structure

Similar to the "sequence" command, this command allows DAS sources to act as reference sources for 3D structures. Clients may request the structure of a given entity, and the source responds with an XML representation of the atomic structure. PDB structures are currently served by a data source maintained by the Wellcome Trust Sanger Institute.

#### Alignment

This command provides a flexible mechanism for exposing pairwise and multiple alignments of entities. As well as full alignments, clients can request partial alignments containing entities within a given range of a query entity. This is particularly useful for clients wishing to display alignments containing large numbers of entities, such as the protein family alignments displayed on the Pfam website [11].

DAS alignments may additionally be used by clients as a means of converting between coordinate systems. For example, the Sanger Institute maintains an alignment DAS source that offers mappings between the UniProt and PDB databases. Using an alignment as an intermediary, it is possible for clients such as SPICE [12] to project features from one coordinate system to the other.

#### Interaction

The interaction command is used for unifying and integrating different sources of molecular interaction data. A DAS source implementing this command supplies XML representations of molecular interactions.

The DAS representation of an interaction is flexible enough to allow many types of interactions, including those for which the interacting region is known and those for which it is not. The XML document contains a list of interactions and a list of the interacting entities (termed "interactors"), with each interaction referencing two or more interactors. In addition to standard attributes such as name and database source, both interactions and interactors may be further described with additional custom properties.

An interaction DAS source can be queried using one or more interactor identifiers, whereupon the DAS source returns interactions involving them all. The client can also request that interactions be filtered by their custom properties, specifying either interactions for which a given property is defined or those for which the property matches a given value.

#### Volmap

The volmap command is used for syndicating 3D structure volume map data from electron microscopy. It accepts a single "query" ID, and the simple XML response contains metadata for the volume map and a link to the raw data. Unlike other DAS commands, the data itself is not encapsulated in XML due to its large size. The 3DEM group at the Spanish National Center for Biotechnology offers DAS reference and annotation servers for volume map data, and have developed the PeppeR client to facilitate its display [13].

#### Sources

The sources command is different from other DAS commands in that it is not implemented by individual DAS sources. Instead it is typically implemented by the servers on which DAS sources are hosted, and provides metadata describing their DAS sources. This allows clients and end users to discover the services a server offers. The command details for each source:

1. The capabilities (commands) the source responds to.

2. The coordinate systems the source offers data for.

3. A contact email address.

4. Custom properties that describe the source further (such as the project the source belongs to).

Through the sources command, the DAS Registry can automatically 'mirror' individual servers, significantly augmenting the federation capabilities of the DAS protocol.

### Protein feature ontology

The DAS protocol is intended to facilitate user-driven data integration such as graphical interfaces, and to enable data providers to quickly and easily expose their data. For these reasons, although the data transport mechanism has a defined structure, unlike other data integration technologies DAS does not impose strict semantic constraints on the data itself. Whilst this has resulted in widespread adoption, data shared via DAS are typically not amenable to automated analysis because the relationships between data types cannot be reliably inferred and it is difficult to assess their relative significance. To address this shortcoming, the DAS/1.53E specification defines an ontology for sharing protein feature annotations within a controlled vocabulary, developed jointly by the BioSapiens, UniProt and Gene Ontology projects. Currently, 34 BioSapiens DAS sources are committed to implementing the ontology in their annotations, though any source may choose to do so.

The ontology is an optional extension to the *features *DAS command, and because it is implemented by convention rather than by modifying the XML schema it is fully backwards compatible. The ontology itself is actually a composite of three ontologies:

1. Sequence Ontology [14], an established ontology describing features of biological sequences.

2. PSI-MOD, an ontology for post-translational modification terms.

3. A new ontology for BioSapiens-specific terms not covered elsewhere, such as literature references and other non-positional annotations.

### Command extensions

The DAS 1.53E specifications defines two new optional extensions to existing commands.

#### Binning

A core principle of DAS is the notion of servers being relatively simple, which lowers the requirements for data providers to expose their data. However, some DAS sources can potentially serve very large numbers of annotation features for a given segment of sequence. This creates problems for user-driven clients that rely on fast response times. Often, the client is not capable of rendering all these features because the user interface has insufficient resolution. For example, a DAS source might annotate every base in a megabase region of the genome, but the user of a graphical client will not be able to see every annotation.

To solve the speed issue, the Ensembl DAS client takes advantage of this fact. It adds a *maxbins *parameter to a "features" command request. This parameter informs the DAS source of the client's maximum available rendering space by means of the number of 'bins' that features may be placed into. The DAS source may then choose to optimise its response by only returning features that are renderable by the client (*i.e. *maximum one per bin). It is up to the DAS source to decide which features it should filter. This process is illustrated in Table 1.

**Table 1:** Binning: illustration of how a DAS source may implement binning.

Positions	11	12	13	14	15		16	17	18	19	20		21	22	23	24	25
Available bins	Bin 1						Bin 2						Bin 3				
					
Annotation score	8	3	9	1	4		7	6	7	2	0		2	4	4	3	5
Features returned			9				7										5

In this contrived example a client requests feature annotations for a segment of sequence between bases 11 and 25, with a *maxbins *parameter of 3. The DAS source has an annotation for every base but, after sorting each into its appropriate bin, returns only those features with the highest scores.

#### Advanced stylesheets

Some DAS sources opt to provide stylesheets – generic blueprints that allow a client, if it so wishes, to render features according to the intention of the DAS source provider. The core specification defines several *glyphs *that a feature can be rendered as such as boxes, lines and arrows. Stylesheets, as in other DAS commands, are provided in XML format and work by specifying the size, colour and type of glyph to be rendered for each type of annotation provided by the features command.

**Figure 1:**
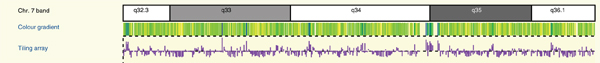
**Advanced stylesheets**. Examples of advanced rendering techniques possible with DAS: colour gradients and tiling arrays, as displayed in Ensembl.

Though stylesheets work well in representing sequence annotations such as exons, it is often desirable for some feature annotations to be rendered in more elegant formats. The 1.53E specification contains new glyph types for the "stylesheet" command that allow a server to define new ways of rendering data. The most notable of these are instructions for rendering plots according to a feature's *score *property. Different plot types include histograms, colour gradients, line plots and tiling arrays (wiggle plots). Figure 1 shows some examples of these formats.

## Implementation

The DAS specification has several client implementations. The Ensembl genome browser [4] incorporates a DAS client for several of its "views", and is able to display data from a wide variety of genomic, gene and protein sequence coordinate systems. It also integrates with SPICE [12], a Java Web-Start application that uses DAS alignments to combine protein sequence and structural annotations. Using SPICE, protein sequence annotations can be projected onto and visualised within a 3D structure. The DASMIweb portal [15] integrates protein-protein and domain-domain interaction datasets. The iPfam website also integrates interaction data, comparing the interaction topologies of different sources by overlaying them in a node graph [16]. Other clients include Dasty [17], a web-based standalone DAS client implemented in Javascript, and the Pfam [11] website.

Several solid client implementations are based on open source libraries, which are available for the Perl and Java programming languages. These include *Bio::Das::Lite *[18] and the *Dasobert *component of BioJava [19]. DAS server implementations are also provided for both languages: ProServer [20] and LDAS [21] for Perl; Dazzle [22] and MyDas [23] for Java.

## Discussion

DAS is a widely adopted protocol for the integration of biological data types in user-driven contexts, commonly used by consortia of distributed institutions such as BioSapiens and ENCODE. Though originally designed for aggregating genomic data, over recent years it has been extended to cover additional data types such as protein structures and molecular interactions. Thus DAS continues to increase its penetration as a data integration platform. The increase in the number of available DAS data sources has necessitated the development of a syndication and discovery service, which was recently established in the form of a DAS Registry. In addition, a Protein Feature Ontology has been developed to fulfil a desire to constrain data to a controlled vocabulary so that it may be treated in a more intelligent manner. Together, these developments are ratified into a new *extended *DAS specification, version 1.53E. This consolidation serves to present a more coherent view of DAS as a flexible data integration platform. The principal strength of DAS lies in the ease with which data providers can expose their data, specifically for visual display. This simplicity makes it a good choice for smaller or experimentally-focussed groups with limited informatics resources wishing to allow their data to be visualised alongside other resources. Its decentralised structure also makes it ideal for clients when integrating frequently changing data. Similarly, since data offered via DAS always adheres to a defined format, changes in data structure are invisible to clients. This is in contrast to other data integration methods such as data warehousing and mediators that wrap individual data sources.

However, other integration strategies do have their advantages. For example, other more complex middleware solutions may offer more advanced querying capabilities or more rigid semantics, which make them more suitable than DAS for data mining or as primary interfaces. Their disadvantages typically lie in the inevitably more involved setup process, textual display and reduced performance. Data warehouses have the capacity to provide high performance and powerful querying for analysis applications, but are often limited by the range of data sources they can integrate. This is due to the resources required to integrate each resource, with imports and structural changes typically being handled by the integrator rather than the data provider.

The DAS protocol will continue to evolve in response to new requirements, enabled largely by the flexibility and simplicity of the original design. Future improvements may include the addition of server-side filtering for sources providing large amounts of data, a *writeback *function for sequence or annotation submission and "come back later" responses for exposing software as a DAS service. Additional commands for new data types such as small molecules are also likely.

## Conclusion

DAS is a data integration mechanism gaining greater popularity in the bioinformatics community, due largely to its simplicity of design. We have expanded the applicability and functionality of the DAS protocol with five new commands, two command extensions and a protein feature ontology. We have consolidated these disparate extensions into a new extended DAS specification, version 1.53E. As a result, DAS now represents a flexible and more coherent data integration platform that spans several areas, from genomic sequences to protein interactions.

## Availability and requirements

• Project home page: 

• Operating system(s): Platform independent

• Programming language: Java, Perl

• Other requirements: Internet Browser

• License: GPL and other open source

• Any restrictions to use by non-academics: none

## Competing interests

The authors declare that they have no competing interests.

## Authors' contributions

AMJ extended and maintains the ProServer library, contributed to the Bio::Das::Lite library, contributed to the Ensembl client, implemented and maintains a variety of DAS servers and wrote the manuscript. MA, HB, RDF, JRM and AP designed the various extensions. MA and HB implemented DASMIweb. TD was involved in the design of the project and is the original author of the Dazzle library. EB and TH contributed guidance throughout the project. RDF is responsible for the Pfam resources and extended the ProServer and Bio::Das::Lite libraries. HH and PJ implemented proteomics DAS resources and the MyDas server library. RCJ implemented Dasty. AK implemented various DAS servers. EK implemented the Ensembl client and server. JRM implemented PeppeR. GAR implemented the ontology. AP co-ordinated the 1.53E specification, implemented SPICE and the DAS registration server and extended and maintains the Dazzle and Dasobert libraries.
